# Rapid Measurement of Total Saponins, Mannitol, and Naringenin in *Dendrobium officinale* by Near-Infrared Spectroscopy and Chemometrics

**DOI:** 10.3390/foods13081199

**Published:** 2024-04-15

**Authors:** Xiangting She, Jing Huang, Xiaoqing Cao, Mingjiang Wu, Yue Yang

**Affiliations:** Zhejiang Provincial Key Laboratory for Water Environment and Marine Biological Resources Protection, College of Life and Environmental Science, Wenzhou University, Wenzhou 325035, China; shexiangting7@163.com (X.S.); jinghuang0224@163.com (J.H.); caoxiaoqing0311@163.com (X.C.); wmj@wzu.edu.cn (M.W.)

**Keywords:** near-infrared spectroscopy, *Dendrobium officinale*, chemometrics, quality evaluation

## Abstract

*Dendrobium officinale* has drawn increasing attention as a dual-use plant with herbal medicine and food applications. The efficient quality evaluation of *D. officinale* is essential to ensuring its nutritional and pharmaceutical value. Given that traditional analytical methods are generally time-consuming, expensive, and laborious, this study developed a rapid and efficient approach to assess the quality of *D. officinale* from different geographical origins by near-infrared (NIR) spectroscopy and chemometrics. Total saponins, mannitol, and naringenin were utilized as quality indicators. Two wavelength selection methods, namely, uninformative variable elimination and competitive adaptive reweighted sampling (CARS), were utilized to enhance the prediction accuracy of the quantification model. Moreover, multiple spectral pretreatment methods were applied for model optimization. Results indicated that the partial least squares (PLS) model constructed based on the wavelengths selected by CARS exhibited superior performance in predicting the contents of the quality indicators. The coefficient of determination (*R_P_*^2^) and root mean square error (*RMSEP*) in the independent test sets were 0.8949 and 0.1250 g kg^−1^ for total saponins, 0.9664 and 0.2192 g kg^−1^ for mannitol, and 0.8570 and 0.003159 g kg^−1^ for naringenin, respectively. This study revealed that NIR spectroscopy and the CARS-PLS model could be used as a rapid and accurate technique to evaluate the quality of *D. officinale*.

## 1. Introduction

*Dendrobium officinale* Kimura et Migo, commonly known as Tiepi Shihu, belongs to the genus *Dendrobium* in the family Orchidaceae [1]. It is a valuable Chinese herbal medicine with a long history and is primarily distributed throughout subtropical and tropical regions, particularly in China, Australia, India, Japan, and other areas [2]. *D. officinale* possesses a diverse range of pharmacological properties, including immunomodulatory effects, anti-fatigue activity, and gastroprotective actions against ulcers; thus, it is called “one of the nine fairy species” [3]. Numerous studies have confirmed the significance of *D. officinale* in the pharmaceutical industry. For instance, Kuang et al. discovered that the polysaccharides in *D. officinale* can effectively reduce blood glucose levels and prevent the onset of diabetes [4]. Zhang et al. revealed that *D. officinale* supplementation can promote alcohol metabolism and alleviate alcoholic fatty liver disease [5]. An increasing body of studies has demonstrated that a variety of active compounds, including polysaccharides, flavonoids, mannitol, naringenin, and total saponins, are closely associated with the antioxidant, anti-cancer, and anti-inflammatory properties of *D. officinale* [6,7,8]. Due to its health benefits and therapeutic properties, *D. officinale* has been widely prepared into a variety of health products, such as oral liquid, lozenges, capsules, and so on. Currently, it is also used as a raw material in drugs such as Granules Dendrobii and Shihu Yeguang pills, which have demonstrated significant therapeutic effects in clinical practice [8]. Wild *D. officinale* resources cannot meet the increasing demand, and most *D. officinale* products available on the market are artificially cultivated. In China, the population of *D. officinale* has led to a continuous increase in its production and turnover, reaching over 27,000 tons and 1.4 billion dollars. In particular, it is widely cultivated in Anhui and Zhejiang Provinces in China [9,10]. However, environmental factors differ across varying geographical origins, causing variations in the content of the active compounds in *D. officinale* that inevitably influence its nutritional and medical values. Some merchants often adulterate or sell substandard products at premium prices, thereby disrupting market integrity and undermining consumer rights. Therefore, the quality evaluation of *D. officinale* is essential. Conventional approaches for identifying *D. officinale* mainly encompass morphological identification [11], fingerprinting [12], and chemical analysis [3]. However, character identification and fingerprinting are inclined to yield insensitive detection results. Moreover, chemical identification, including ultraviolet–visible spectroscopy (UV-vis), high-performance liquid chromatography (HPLC), and gas chromatography, is time-consuming, laborious, and expensive; they also require complex sample pretreatment and skilled operators [13]. Thus, a rapid and efficient method for the measurement of active components in *D. officinale* is needed.

Near-infrared (NIR) spectroscopy is an ideal choice due to its benefits such as fast analysis speed, high accuracy, easy operation, absence of additional reagents, and environmental friendliness [14]. It is now extensively utilized in various fields, including pharmaceuticals, petrochemicals, textiles, cosmetics, and medical applications [15]. NIR is a type of light that falls between the visible range and the mid-infrared range. However, the NIR spectrum is characterized by complex and overlapping peaks and useless information from background and noise, resulting in difficulty in clearly distinguishing the specific spectral range corresponding to the biochemical substance. To enhance model accuracy, researchers commonly employ spectral pretreatment methods such as standard normal variable (SNV) transformation, Savitzky–Golay (SG) smoothing, first derivative (1D), second derivative (2D), and multivariate scattering correction (MSC) to remove noise interference, linearity correction, and spectral fitting [16]. Wavelength selection methods are crucial for feature extraction. Uninformed variable elimination (UVE) [17], competitive adaptive reweighted sampling (CARS) [18], and the non-information variable elimination algorithm are widely accepted methods for wavelength selection, and each possesses unique strengths in various aspects. The saponins, considered important components of multiple traditional Chinese medicines, possess numerous medicinal properties such as immune activity, hemolytic activity, antimicrobial effects, antiviral effects, and anti-cancer properties [19]. Mannitol is a sugar-free and functional sweetener that can control cell expansion and scavenge free radicals, making it suitable for use as a food additive to enhance the nutritional value of food [20]. Naringenin, a commonly consumed flavonoid substance, has been widely used in clinical practice due to its anti-inflammatory and anti-infective medicinal properties [21]. Until now, the determination of active components in *D. officinale* through NIR spectroscopy has been scarcely studied. Ma et al. used NIR spectroscopy to predict the total polyphenol content and antioxidant activity in *D. officinale* [22]. Yun et al. developed a green method based on NIR spectroscopy to quantify the polysaccharides *in D. officinale* [23]. The quantification indicators of the previous studies mainly focused on polysaccharides and polyphenols. To the extent of our knowledge, the rapid measurement of total saponins, mannitol, and naringenin in *D. officinale* via NIR spectroscopy has not been conducted.

This work aimed to investigate the potential of NIR spectroscopy and chemometrics as a fast and convenient method for determining the total saponins, naringenin, and mannitol in *D. officinale*. To achieve this objective, we investigated the prediction performance of three NIR quantitative models. Subsequently, multiple spectrum pretreatment techniques and wavelength selection methods were examined to optimize model performance. Using NIR spectroscopy to establish a quantitative model can achieve quick detection of total saponins, naringenin, and mannitol in *D. officinale*, thereby reducing the analysis time and enhancing efficiency. 

## 2. Materials and Methods

### 2.1. Materials and Reagents

A total of 120 *D. officinale* samples were purchased from various provinces in China through online purchasing platforms. After being dried to a consistent weight, the materials were ground into a fine powder and sifted through an 80-mesh screen. The resulting powder samples were then stored in sealed and opaque plastic containers until analysis.

Deionized water was generated using a Milli-Q system (Millipore, Burlington, MA, USA). Analytical-grade sodium periodate, hydrochloric acid, L-rhamnose acetate, acetic acid, acetylacetone, methanol, perchloric acid, vanillin, and anhydrous ethanol were procured from Sinopharm Chemical Reagent Co., Ltd. (Shanghai, China). Neutral alumina was also of analytical grade. High-purity standards of ginsenoside Re (≥98%), mannitol (≥98%), and naringenin (≥98%) were purchased from Must Biological Technology Co., Ltd. (Chengdu, China). HPLC-grade methanol and phosphoric acid were obtained from Sinopharm Chemical Reagent Co., Ltd. (Shanghai, China). 

### 2.2. NIR Spectral Acquisition

The Antaris II FT-NIR spectrometer (Thermo Fisher Scientific, St. Louis, MO, USA) was utilized for NIR spectral acquisition. A quartz sample cup was filled with approximately 2 g of the material prior to acquiring the NIR spectrum. The spectrum was obtained at an interval of 3.854 cm^−1^ and covered a wavelength range of 1000–2500 nm (10,000–4000 cm^−1^). With air serving as the reference, each spectrum was scanned 64 times. Each sample was scanned three times, and the average spectrum was utilized for subsequent analysis.

### 2.3. Reference Assays

The contents of total saponins, mannitol, and naringenin in *D. officinale* were determined by the macroporous adsorption resin method [24], the sodium periodate–hydrochloric acid method [25], and the methanol-heated reflux–HPLC method [26], respectively. The corresponding standard reference materials were ginsenoside Re, D-mannitol, and naringenin.

#### 2.3.1. Determination of Total Saponins

An amount of 0.1 g of sample powder was accurately weighed and placed in a 5 mL centrifuge tube. About 2 mL of 70% anhydrous ethanol was added, shaken well, ultrasonically extracted for 90 min, and cooled overnight to obtain the sample solution. The chromatographic tube was fixed vertically with a 5 mL syringe. The D101 microporous resin was packed to a height of 3 cm, followed by the addition of neutral alumina to a height of 1 cm. The column was washed with 25 mL of 70% ethanol, and the eluent was discarded. About 1 mL of the treated sample solution was carefully added to the upper part of the column. The column was washed with 25 mL of deionized water to wash away water-soluble impurities such as sugar, and the eluent was discarded. Finally, 25 mL of 70% ethanol was used to perform the elution to extract the total saponins. The eluent was collected in an evaporating dish and dried at 60 °C in an oven for subsequent use. Subsequently, 1 mL of perchloric acid–5% vanillin–glacial acetic acid solution was added to the dried evaporating dish, which was rotated to dissolve any remaining residue. The resulting mixture was transferred into a centrifuge tube with a lid (10 mL), and the lid was placed in a water bath at 60 °C for 15 min. After the precise addition of 5 mL of glacial acetic acid and thorough agitation, the absorbance was measured at 560 nm by using a UV-vis spectrophotometer (UV-1810, Puxi, Shanghai, China). Each sample was measured three times, and the average was used for further analysis.

#### 2.3.2. Determination of Mannitol

An amount of 10 mL of deionized water was mixed with 0.25 g of the sample powder and extracted for 2 h by continuous reflow at 90 °C. The solution underwent filtration, and the residue was washed with 2 mL of deionized water three times. The replenished filtrate and lotion were combined in a 25 mL volumetric flask. Deionized water was added to the scale in a constant volume to obtain the test solution. We accurately pipetted 1 mL of the aforementioned test solution into a test tube, followed by the addition of 1 mL of sodium periodate and hydrochloric acid solution. After reacting for 10 min at room temperature, the solution was then added to 2 mL of 0.1% rhamnose solution and 4 mL of Nash reagent. Finally, the mixture was heated at 53 °C for 15 min. The UV-vis spectrophotometer was utilized to measure the sample at 413 nm. Each sample was measured in triplicate to minimize errors. Each sample was measured three times, and the average was used for further analysis.

#### 2.3.3. Determination of Naringenin

An amount of 0.1 g of sample powder was dissolved in 2 mL of methanol and extracted for 2 h through heating reflux at 70 °C. The solution was dried in a water bath and then filtered through a 0.45 µm microporous filter membrane. The residue was similarly filtered with 0.5 mL of methanol. Subsequently, all the filtrates were preserved in a refrigerator (4 °C) until use. Finally, the naringenin content in the filtrate was determined by using an Agilent 1200 HPLC system (Agilent Technologies, Santa Clara, CA, USA). The component separation was achieved using a Waters Xbridge-C18 column (4.6 mm × 250 mm, 5 μm) (Waters Corporation, Milford, MA, USA). The main methods were as follows: The mobile phase consisted of 0.2% phosphoric acid (A) and methanol (B). The gradient elution modes were 25% B at 0–5 min and 25–30% B at 5–10 min. The flow rate was set to 1.0 mL min^−1^, and the column temperature was 25 °C. Additionally, the injection volume was set to 20 μL, and the ultraviolet spectrum of the sample was set at 290 nm. Each sample was measured three times, and the average was used for further analysis.

### 2.4. Chemometrics

#### 2.4.1. Spectral Data Processing

Various spectral preprocessing algorithms are frequently employed to mitigate random noise and disorder variations in the spectral data that stem from factors unrelated to the characteristics of the sample. The commonly used methods include MSC, SG smooth, 1D + SG, SNV, and 2D + SG. Derivatives are the most frequently used approach to eliminating overlapping peak effects [27]. SNV and MSC are often applied to remove multiplicative impacts, which will somewhat weaken the influence of sample particle size or scattering effect on spectral data [28]. The details of these algorithms are described below. SG smoothing is widely employed as a denoising method, which primarily employs the averaging of multiple measurements to reduce noise and enhance the signal-to-noise ratio in scenarios where the spectrum contains zero-mean random white noise. The average value after smoothing at wavelength *k* is as follows:

(1) $$x_{k,smooth}={\bar{x}}_{k}=\frac{1}{H}\sum_{i=-w}^{+w}x_{k+i}h_{i}$$

where *h_i_* and *H* are the smoothing factor and the normalization factor, respectively; w is the width of the smoothing window; and *i* = 1, 2, …, w.

The first derivative and second derivative are commonly used pretreatment methods for baseline correction and identification of overlapping spectral differences. For a spectrum at wavelength *k* with a gap size g, the first and second derivative spectra can be respectively calculated as follows:

First derivative:

(2) $$x_{k,1st}=\frac{x_{k+g}-x_{k-g}}{g}$$

Second derivative:

(3) $$x_{k,2nd}=\frac{x_{k+g}-2x_{k}+x_{k-g}}{g^{2}}$$

MSC is used to eliminate scattering interference caused by particle size and other factors and has been widely used in NIR analysis. For a spectrum of data *X*, its algorithm steps are as follows:

(1) The average spectrum of the calibration sample was calculated.
(2) The linear regression between the spectrum of each calibration sample and the average spectrum was performed.

(4) $$X=m_{i}\bar{X}+b_{i}$$

where *m_i_* and *b_i_* are the regression coefficients and $\bar{X}$ is the mean of the spectrum.

(3) MSC transforms the spectrum as follows:

(5) $$X_{\left(MSC\right)}=\frac{x-b_{i}}{m_{i}}$$

The SNV method is probably the second most commonly applied approach for scatter correction in spectrum analysis. Its primary purpose is to mitigate the multiplicative effects caused by scattering and solid particle size. Each spectrum is centered, then scaled by dividing by its standard deviation.

(6) $$X_{i,j}^{SNV}=\frac{x_{i,j}-\bar{x}}{\sqrt{\frac{\sum_{k=1}^{m}{\left(x_{k}-\bar{x}\right)}^{2}}{\left(m-1\right)}}}$$

where $\bar{x}$ is the mean of the spectrum; *x_i,j_* is the corresponding original element of the spectrum *i* at variable *j*; *m* is the number of wavelengths in the spectrum; and *k* = 1, 2, …, m.

#### 2.4.2. Wavelength Selection Methods

In the original data, redundancy information exists between adjacent wavelengths, which has a negative effect on the accuracy and stability of calibration models. As such, appropriate methods must be employed to extract the useful wavelengths in spectral analysis.

The UVE method is a commonly employed technique for ranking the importance of variables based on their regression coefficients [29]. UVE first introduces a set of random noise matrices and then constructs a partial least squares (PLS) model through cross-validation. The ratio between the mean regression coefficient and its standard deviation for each variable in the coefficient matrix is calculated. Subsequently, the maximum ratio of noise matrices is used as a threshold to eliminate irrelevant information with ratios below this threshold from spectral lines. The relationship can be expressed as follows:

(7) $$h_{i}=\frac{mean(\beta_{i})}{std(\beta_{i})}$$

where *h_i_* is the ratio of the mean(β_i_) and std(β_i_); mean(β_i_) is the mean value of the regression coefficient of variable *i*; and std(β_i_) is the standard deviation of the regression coefficient of variable *i*.

**Figure 1:**
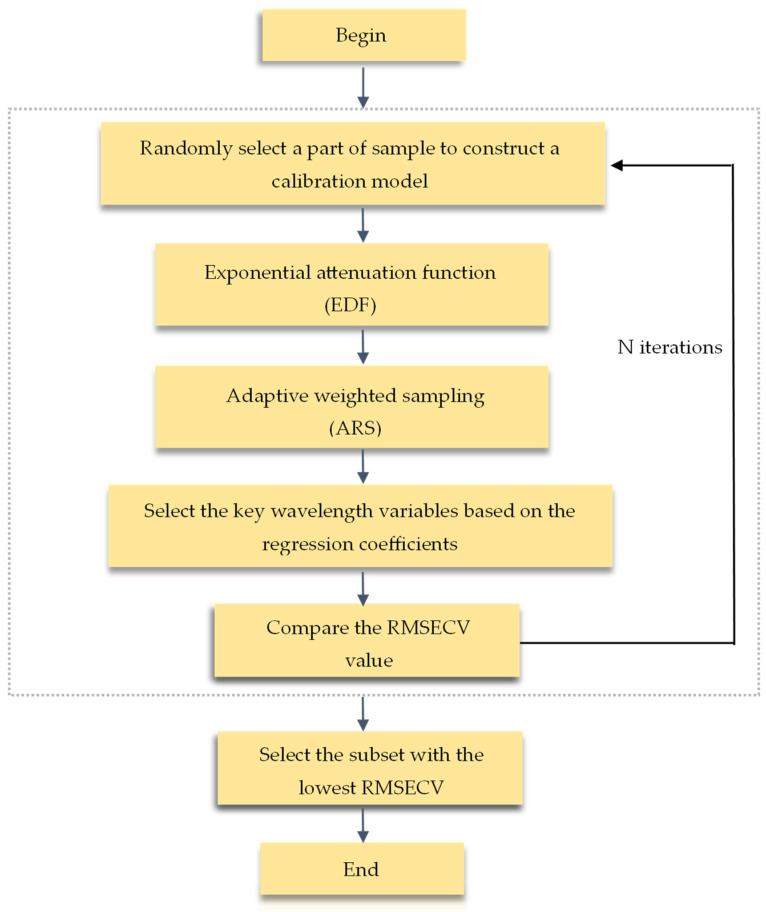
Flow chart of the competitive adaptive reweighted sampling (CARS) algorithm.

CARS is a feature variable selection method combining Monte Carlo sampling and regression coefficients of the PLS model, which imitates the “survival of the fittest” concept of Darwin’s theory [30]. Figure 1 presents the scheme of the CARS algorithm. Initially, a part of the sample was randomly selected from the correction set for PLS modeling, and random modeling was repeated several times. The exponential attenuation function (EDF) was used to remove wavelengths with a small weight of regression coefficient. After multiple modeling, the wavelengths with a large absolute weight of regression coefficient were screened by adaptive weighted sampling (ARS), and the subset of the generated new variables was used for PLS modeling analysis. For mannitol and naringenin, the PLS model inputs were a data matrix with dimensions of 120 × 1557. As for total saponins, three samples were excluded due to the abnormal concentrations; thus, the PLS model input matrix size was 117 × 1557. Finally, cross-validation was performed to select the subset with the lowest root mean square error of cross-validation (RMSECV) value, which was the best combination of wavelength variables [31].

### 2.5. Model Performance Evaluation

In terms of quantitative analysis, the performance evaluation of the calibration model depends on several indicators, including the coefficient of determination of prediction (*R_p_*^2^), the root mean square error of prediction (*RMSEP*), the coefficient of determination of calibration (*R_c_*^2^), and the root mean square error of calibration (*RMSEC*) [32]. In general, a calibration model is considered satisfactory if *R_c_*^2^ and *R_p_*^2^ are close to 1 and *RMSEC* and *RMSEP* are small [33].

These parameters were calculated as follows:

(8) $$R_{C}^{2}=1-\frac{\Sigma {\left(C_{i}-{\hat{C}}_{i}\right)}^{2}}{\Sigma {\left(C_{i}-C_{n}\right)}^{2}}$$

(9) $$R_{P}^{2}=1-\frac{\Sigma {\left(C_{i}-{\hat{C}}_{i}\right)}^{2}}{\Sigma {\left(C_{i}-C_{m}\right)}^{2}}$$

(10) $$RMSEC=\sqrt{\frac{\Sigma {\left({\hat{C}}_{i}-C_{i}\right)}^{2}}{n}}$$

(11) $$RMSEP=\sqrt{\frac{\Sigma {\left({\hat{C}}_{i}-C_{i}\right)}^{2}}{m}}$$

where *C_i_* is the *i*-th measured value of the reference method; ${\hat{C}}_{i}$ is the *i*-th predicted value of the model; *C_n_* is the mean value of the measured values of *n* samples; *C_m_* is the mean of the predicted values of m samples; *m* is the number of samples in the prediction sets; and *n* is the number of samples in the calibration sets.

In this work, all data analyses, including spectral pretreatment, wavelength selection, and PLS modeling, were performed in the MATLAB software (2014a, Mathworks Inc., Natick, MA, USA).

## 3. Results and Discussion

### 3.1. Spectral Feature Analysis

**Figure 2:**
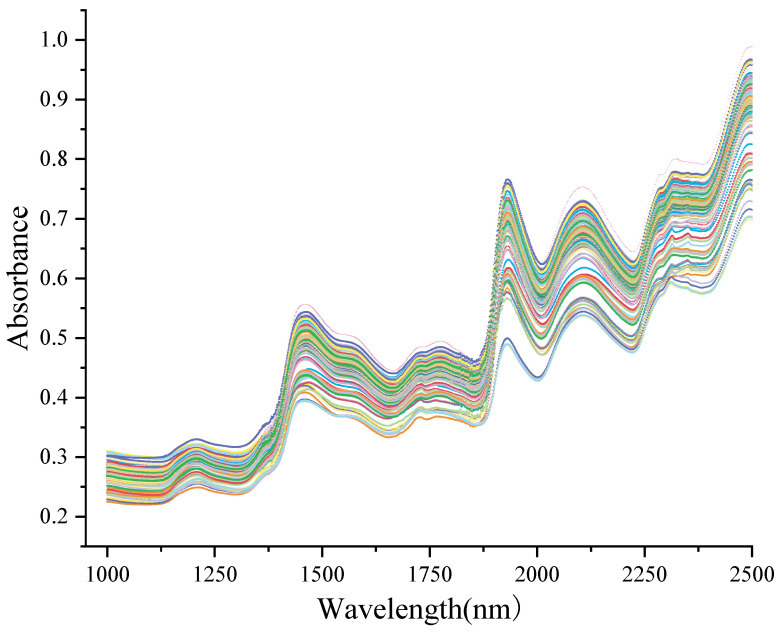
Raw near-infrared spectra of all 120 *Dendrobium officinale* samples. Each line represents the near-infrared spectrum of each sample.

The NIR absorption spectra of all *D. officinale* samples within 1000–2500 nm are shown in Figure 2. The absorption peaks near 1500, 1940, 2150, and 2315 nm were relatively strong. The intense absorption peak near 1500 nm was attributed to the first –OH overtone and the first –NH overtone [34]. Additionally, the absorption peak at about 1940 nm was associated with the stretching vibration of the –OH group [35]. The peaks observed at 2150 and 2315 nm mainly came from the –CH stretching vibrations in the benzene ring and –CH bending vibrations, respectively [36].

### 3.2. Outlier Detection and Sample Partition

Mahalanobis distance aims to calculate the distance between the average spectrum of the sample sets and each sample spectrum, and it is commonly used for identifying outliers [37]. The results showed that no spectra errors were found for mannitol and naringenin, but three samples for total saponins were identified as spectra errors and removed. Thus, for total saponins, mannitol, and naringenin, a total of 117, 120, and 120 samples were used for subsequent model development. Moreover, the calibration and prediction sets must be appropriately divided. The calibration set serves as the foundation for model establishment, and the prediction set is used to evaluate model performance. In this study, the Kennard and Stone (KS) algorithm was used for sample partition. The principle of the KS algorithm is to calculate the Euclidean distance between the remaining and selected samples. The two samples with the maximum and minimum distances were found and included in the calibration set. This process was then repeated until the desired number was reached [38,39]. The calibration and prediction sets consisted of 96 and 24 samples for mannitol, 96 and 24 samples for naringenin, and 94 and 23 samples for total saponins, respectively. Thus, for mannitol and naringenin, a data matrix with dimensions of 96 × 1557 was used for model calibration, while a matrix of 24 × 1557 was used for model validation. As for total saponins, a data matrix with dimensions of 94 × 1557 was used for model calibration and 23 × 1557 for model validation. The statistical results of samples in the calibration and prediction sets are presented in Table 1, including minimum values, maximum values, and means. In the calibration set samples, the range of reference values for these three quality parameters almost covered the reference ranges of the prediction set. Thus, the sample partition was reasonable, contributing to a high-precision model.

### 3.3. PLS Models Based on Various Wavelength Selection Methods

After appropriate segmentation, a PLS regression model was established using NIR spectral data and the corresponding output targets. In this study, three different PLS models, namely, full-PLS, UVE-PLS, and CARS-PLS, were developed and compared to evaluate the model performance for total saponins, mannitol, and naringenin. Among them, the full-PLS model was developed based on the full spectrum data, whereas the UVE-PLS and CARS-PLS models were established using wavelength variables selected by the UVE and CARS algorithms, respectively.

#### 3.3.1. Results of the Full-PLS Model

The full-PLS model is a PLS regression model developed using the full spectrum (1000–2500 nm), and it is commonly adopted as a benchmark to evaluate the accuracy of chemometrics. Given that the data were measured at an interval of 0.8 nm over the full spectral range, the full-PLS model contained 1557 data points. The PLS model was constructed with a 120 × 1557 matrix as input, and the corresponding component of interest was utilized as output. However, the prediction ability of the PLS model is negatively impacted by significant scatter in the raw spectra, which may be caused by varying sample sizes and the noise of the instrument. Therefore, in this study, five different spectral pretreatment methods were used to improve the performance of the model, namely, smooth, MSC, 1D + SG, SNV, and 2D + SG. The optimal results of different spectral pretreatment methods on the full-PLS model are listed in Table 2. The results indicated that the NIR spectra data processed by smooth MSC and SNV exhibited good performance for total saponins, mannitol, and naringenin, respectively. Based on the preprocessed spectra, the different PLS models were constructed, and their results are listed in Table 3. For mannitol, the full-PLS model yielded satisfactory outcomes with *R_C_*^2^ and *R_P_*^2^ values exceeding 0.9. As for total saponins and naringenin, the full-PLS model performed on the full spectrum resulted in calibrations of *R_C_*^2^ = 0.9494 with *RMSEC* = 0.1421 g kg^−1^ and *R_C_*^2^ = 0.8589 with *RMSEC* = 0.002715 g kg^−1^, respectively. The prediction set for total saponins and naringenin led to *R_P_*^2^ = 0.8506 with *RMSEP* = 0.1439 g kg^−1^ and *R_P_*^2^ = 0.8432 with *RMSEP* = 0.003195 g kg^−1^, respectively. For both quality parameters, a *R_P_*^2^ of 0.8 was obtained, which was lower than 0.9, indicating that the prediction performance of the full-PLS model was not good. The full-range spectrum contained a significant number of irrelevant spectral wavelength variables, which possibly weakened the model’s performance. Therefore, in this study, UVE and CARS wavelength selection methods were used to enhance model prediction accuracy.

#### 3.3.2. Results of the UVE-PLS Model

**Figure 3:**
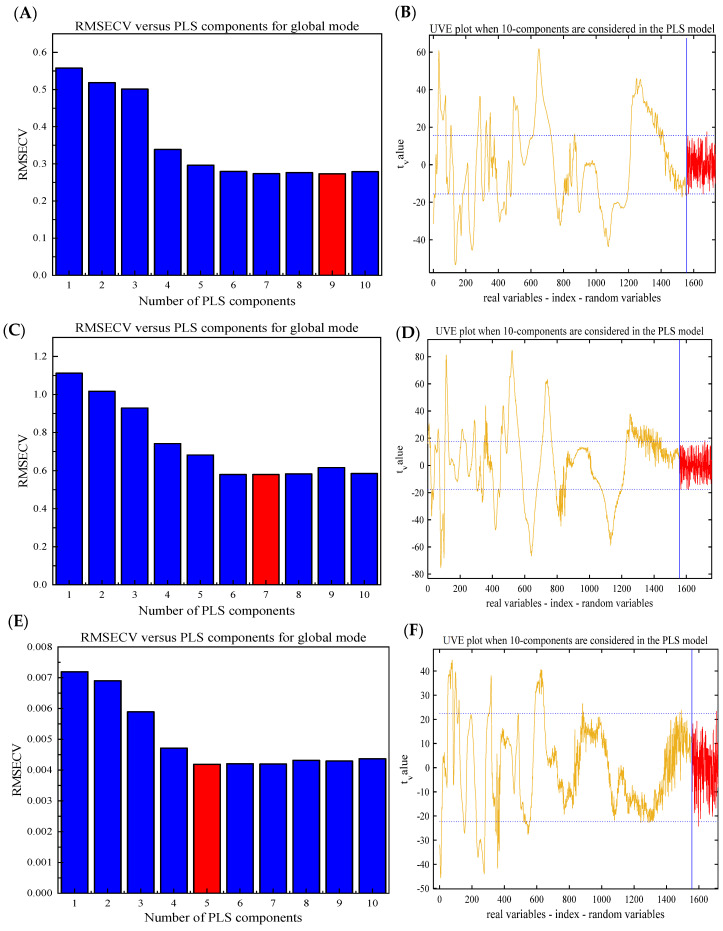
Root mean square error of cross-validation (*RMSECV*) versus partial least squares (PLS) factors for total saponins (**A**), mannitol (**C**), and naringenin (**E**). Stability distribution curve of the uninformed variable elimination (UVE) method for total saponins (**B**), mannitol (**D**), and naringenin (**F**). The red boxes represent the optimal number of PLS components corresponding to the lowest *RMSECV* values. The blue longitudinal straight line indicates the boundary between wavelength variability and noise variability at 1557 nm. The yellow line represents true variations in wavelength, whereas the red line comprises added random noise. The blue horizontal dashed lines are the thresholds based on the random number added.

UVE was used for variable screening to eliminate non-measured sample information in the NIR spectrum. Only specific band spectral variables were collected, thereby simplifying overlapping peaks and complex spectra and shortening the detection time [40]. Figure 3B,D,F show the stability coefficients of the total saponins, mannitol, and naringin in *D. officinale* at each wavelength point, respectively. The longitudinal straight line precisely delineated the boundary between wavelength variability and noise variability at 1557 nm. The region to the left of this boundary represented true variations in wavelength, whereas the region to its right comprised added random noise. The horizontal dashed lines in the graph determined the threshold based on the random number added. The variable between the two lines was considered an uninformative variable and needed to be removed. Ultimately, a total of 872, 767, and 256 variables were selected for total saponins, mannitol, and naringin, respectively. In UVE-PLS, the original spectral data were compressed by principal component analysis (PCA), and a large amount of sample information can be interpreted by a new set called principal components (PCs). More PCs tend to cause “overfitting” problems, leading to good predictions for calibration samples but poor predictions for prediction samples [41]. A few PCs often produce an “underfitting” model, which has poor predictions for both calibration and prediction samples. As such, finding the appropriate number of PCs is crucial for constructing a PLS model with high prediction accuracy. Herein, a cross-validation procedure was used to determine the optimal number, and the changes in *RMSECV* values with the number of PCs are described in Figure 3A,C,E. Finally, 9, 7, and 5, which yielded the lowest *RMSECV* values, were identified as the optimal numbers of PCs for total saponins, mannitol, and naringenin, respectively. Table 3 presents the results of UVE-PLS. Compared with the full-PLS model, the UVE-PLS model showed better performance for mannitol because *R_P_*^2^ increased from 0.9385 to 0.9437 and *RMSEP* decreased from 0.2876 kg^−1^ to 0.2795 kg^−1^. However, for total saponins and naringenin, the UVE-PLS model performed poorly in the prediction set (*R_P_*^2^ = 0.8210, *RMSEP* = 0.1623 g kg^−1^ for total saponins; *R_P_*^2^ = 0.8326, *RMSEP* = 0.003390 g kg^−1^ for naringenin).

#### 3.3.3. Results of the CARS-PLS Model

**Figure 4:**
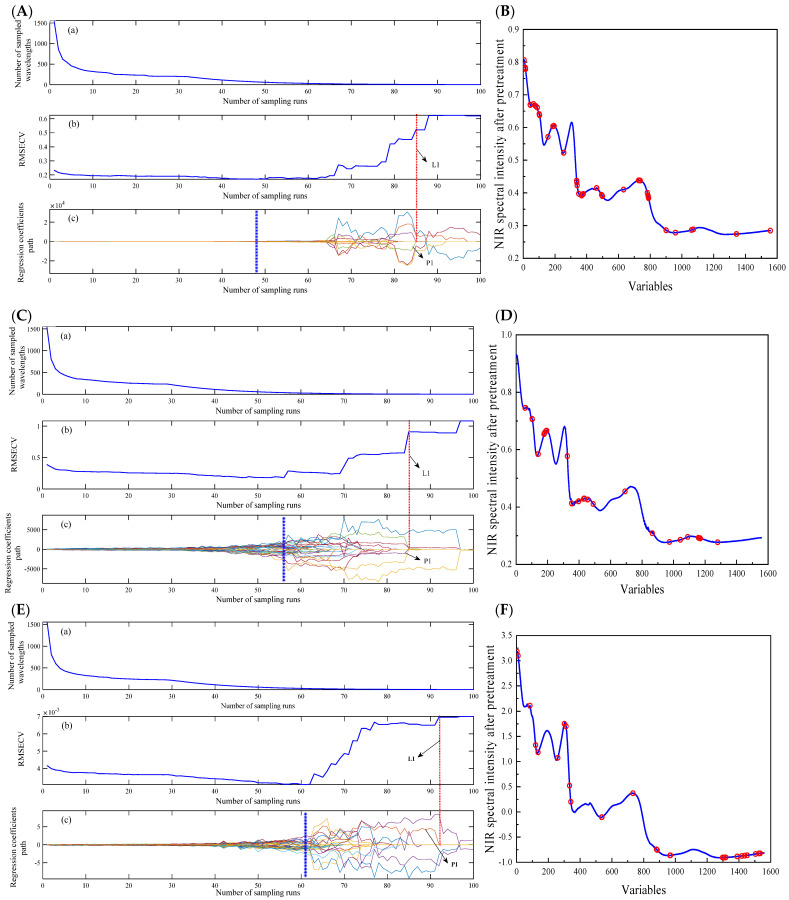
Plot of CRAS wavelength selection and distribution on spectra data for total saponins (**A**,**B**), mannitol (**C**,**D**), and naringenin (**E**,**F**).

The CARS algorithm is often used in conjunction with PLS models to identify informative wavelengths, thereby reducing computational complexity and creating high-performance calibration models. It employs EDF and ARS to identify the wavelengths with the highest absolute regression coefficients. During the execution of CARS, 100 iterations of Monte Carlo sampling were performed, resulting in 100 subsets with different wavelengths. A cross-validation procedure was used to evaluate the subsets, and the key wavelengths were identified as those present in the subset with the lowest *RMSECV* value. The process of wavelength selection by CARS is illustrated in Figure 4A,C,E. As shown in Figure 4E (using naringenin as an instance), the changes in the number of sampled wavelengths (a), *RMSECV* values (b), and regression coefficient paths for each wavelength (c) were obvious as the sampling runs increased. In Figure 4E(a), there was a significant decrease in the number of sampled wavelengths as the number of sampling runs increased from 0 to 10, followed by a gradual change. This suggests that the CARS selection process included both initial screening and subsequent refinement stages. *RMSECV* showed a gradual decrease due to the elimination of irrelevant wavelength variables, reaching its minimum value at sampling run 61, as indicated by the blue asterisk line in Figure 4E(c). Combined with Figure 4E(c), the significance of key wavelengths could be illustrated. Specifically, a key wavelength denoted as P1 was obtained with a regression coefficient equal to zero, indicating that this key wavelength was eliminated. As shown in Figure 4E(b) (marked by the dotted line L1), *RMSECV* immediately increased, indicating that the performance of the model decreased. Finally, CARS selected 66, 39, and 28 key wavelengths for total saponins, mannitol, and naringenin, respectively. The distribution of wavelength variables on the full range of the spectrum is presented in Figure 4B,D,F. The results of the CARS-PLS model are listed in Table 3. The optimal numbers of PCs for the CARS-PLS model were determined to be 16, 17, and 17 for total saponins, mannitol, and naringenin, respectively. As shown in Table 3, the CARS-PLS results for total saponins were *R_C_*^2^ = 0.9626 and *R_P_*^2^ = 0.8949, *RMSEC* = 0.1221 g kg^−1^, and *RMSEP* = 0.1250 g kg^−1^; the CARS-PLS results for mannitol were *R_C_*^2^ = 0.9868 and *R_P_*^2^ = 0.9664, *RMSEC* = 0.1307 g kg^−1^, and *RMSEP* = 0.2192 g kg^−1^; and the CARS-PLS results for naringenin were *R_C_*^2^ = 0.8888 and *R_P_*^2^ = 0.8570, *RMSEC* = 0.002411 g kg^−1^, and *RMSEP* = 0.003159 g kg^−1^.

#### 3.3.4. Discussion of Results

**Figure 5:**
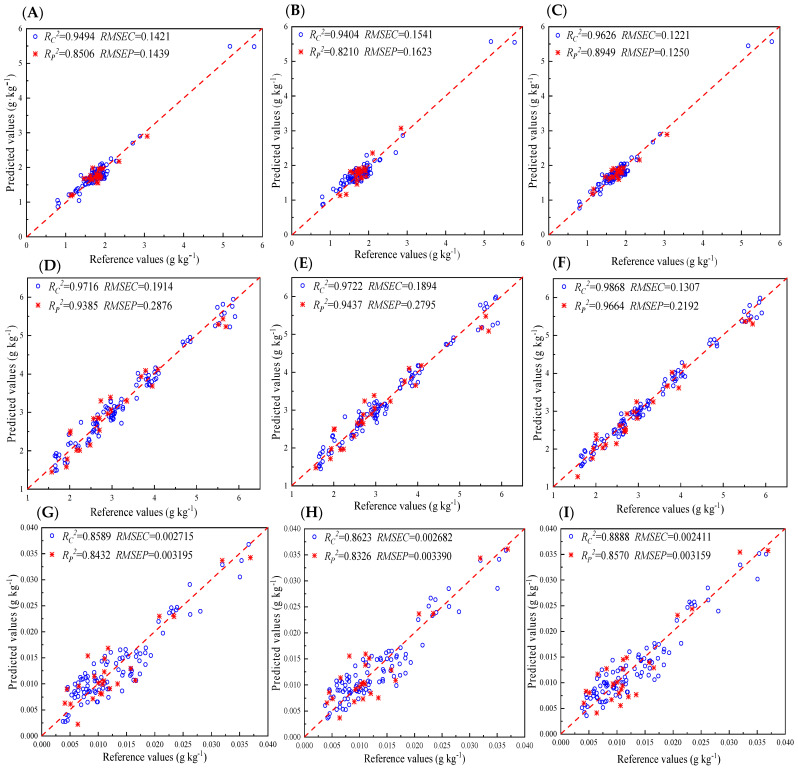
Reference values versus predicted values for total saponins (**A**–**C**), mannitol (**D**–**F**), and naringenin (**G**–**I**) using full-PLS models (**A**,**D**,**G**), UVE-PLS models (**B**,**E**,**H**), and CARS-PLS models (**C**,**F**,**I**).

Different PLS models exhibited varying predictive capabilities for the three quality indicators. For total saponins and naringenin, the prediction accuracy of the three PLS models could be arranged in the following order: UVE-PLS < full-PLS < CARS-PLS. For mannitol, the performance of the PLS models was in the following order: full-PLS < UVE-PLS < CARS-PLS. CARS-PLS was superior to the two other PLS models in predicting total saponins, mannitol, and naringenin, with the highest *R_P_*^2^ and lowest *RMSEP* values. The CARS method can select wavelength variables with high weights and remove wavelength variables with low weights to improve model performance [42]. Although the UVE method is effective in eliminating irrelevant variables and preventing the model from overfitting, it retains too many variables, which might lead to poor model prediction performance [43]. In addition, for total saponins and naringenin, the performance of the model was degraded after the UVE method was processed. This result suggested that some key wavelengths may have been eliminated by UVE during variable selection. The scatter plots of full-PLS models (A, D, and G), UVE-PLS models (B, E, and H), and CARS-PLS models (C, F, and I) for total saponins (A, B, and C), mannitol (D, E, and F), and naringenin (G, H, and I) are presented in Figure 5. It illustrates the correlation between NIR prediction values and reference measured values, and the red dashed line (1:1) represents the ideal results. Overall, the CARS-PLS models demonstrated satisfactory performance in predicting total saponins, mannitol, and naringenin. The good potential of the CARS wavelength selection method for model improvement has been demonstrated in numerous studies. For example, Cao et al. employed NIR spectroscopy and three wavelength selection methods, including synergy interval (SI), genetic algorithm (GA), and CARS, to quantify the polyphenol content of *Sargassum fusiforme*. The results showed that CARS-PLS achieved more satisfactory outcomes with a *RMSEP* of 3.23 g kg^−1^ and a *R_P_*^2^ of 0.99 in an independent prediction set, respectively [44]. Guo et al. used successive projection algorithms (SI, GA, and CARS) to optimize the prediction models of soluble solid content. It was found that CARS-PLS achieved the highest prediction accuracy, with *R_P_* and *RMSEP* values of 0.9808 and 0.327 °Bx, respectively [45]. Guo et al. utilized SI, GA, and CARS to select feature variables and constructed PLS models for predicting the plaque area of apples. The results indicated that the PLS model, based on the wavelengths selected by the CARS algorithm, obtained the best performance [46]. Jiang et al. confirmed that combinations of NIR spectroscopy with three distinct wavelength selection methods, namely variable combination population analysis, variable iterative space shrinkage approach, and CARS, could effectively predict aflatoxin B1 levels in wheat. It was found that the CARS exhibited superior overall prediction performance, yielding an *RMSEP* value of 2.0965 ug kg^−1^ and an *R_P_*^2^ value of 0.9935 [47]. The superior predictive performance of the CARS-PLS model can also be demonstrated by the lower RMSECV values and higher residual predictive deviation (RPD) values (Table 3). Herein, the RMSECV values were obtained by using the leave-one-out cross validation (LOOCV) method. In LOOCV, the samples in the calibration set are selected one by one, with the others used to construct the model and the selected sample employed for validation. Therefore, all the calibration samples can be predicted once. The CARS-PLS model achieved RMSECV of 0.1799, 0.0031, and 0.1691 for mannitol, naringin, and total saponins, respectively, which was lower than other PLS models. RPD, which is defined as the ratio of the standard deviation of the reference values to the RMSEP, was also used to assess the model’s performance. The higher the RPD, the better the model’s performance. Specifically, RPD > 2 indicates exceptional performance; 1.4 < RPD < 2 represents general performance; and RPD < 1.4 represents poor performance [48]. CARS-PLS got the best performance with the highest RPD, which were 5.26 for mannitol, 2.51 for naringin, and 2.92 for total saponins, respectively, indicating that the CARS-PLS model performed well in the prediction of mannitol, naringin, and total saponins in *D. officinale*.

## 4. Conclusions

In this study, a promising CARS-PLS model was established and compared with full-PLS and UVE-PLS for its predictive performance and model robustness. Among the three PLS models, the CARS-PLS model exhibited the most satisfactory performance for the three quality indicators in *D. officinale*. Compared with the full-PLS model, *RMSEP* of the CARS-PLS model decreased by 13.13%, 23.78%, and 1.12% for total saponins, mannitol, and naringenin, respectively. These results demonstrated that the wavelength selection procedure could effectively enhance the prediction performance of the model. Overall, the results confirmed the feasibility of rapid measurement of total saponins, mannitol, and naringenin in *D. officinale* via NIR spectroscopy.

## Figures and Tables

**Table 1:** Statistical results of all samples in the sample sets.

Parameter	Calibration (g kg^−1^)			Prediction (g kg^−1^)		
	Minimum	Maximum	Average	Minimum	Maximum	Average
Total saponins (117)	0.79	5.79	1.81	1.13	3.07	1.79
Naringenin (120)	0.0038	0.0366	0.0130	0.0041	0.0369	0.0129
Mannitol (120)	1.65	5.91	3.24	1.57	5.69	3.09

**Table 2:** Effects of different spectra of pretreatments on full-partial least squares (PLS) models.

Parameter		Raw	Smooth	1D + SG	2D + SG	MSC	SNV
Total saponins	*R*_C_^2^	0.9480	0.9494	0.9246	0.9082	0.9513	0.9401
	*R*_P_^2^	0.8421	0.8506	0.2626	0.0704	0.7343	0.7205
	RMSEC (g kg^−1^)	0.1441	0.1421	0.1739	0.1978	0.1414	0.1567
	RMSEP (g kg^−1^)	0.1464	0.1439	0.2272	0.2056	0.1441	0.1446
Mannitol	*R*_C_^2^	0.8960	0.9444	0.9282	0.9579	0.9716	0.9517
	*R*_P_^2^	0.8682	0.9233	0.7984	0.5476	0.9385	0.9303
	RMSEC (g kg^−1^)	0.3509	0.2565	0.3073	0.2402	0.1914	0.2495
	RMSEP (g kg^−1^)	0.4822	0.3704	0.5303	0.7035	0.2876	0.3073
Naringenin	*R*_C_^2^	0.8053	0.8012	0.9236	0.8828	0.8581	0.8589
	*R*_P_^2^	0.8298	0.8313	0.2245	0.1377	0.8415	0.8432
	RMSEC (g kg^−1^)	0.003117	0.003150	0.002149	0.002679	0.002723	0.002715
	RMSEP (g kg^−1^)	0.003529	0.003515	0.003839	0.003938	0.003202	0.003195

**Table 3:** Results of the PLS models based on different wavelength selection methods.

Parameter	Model	PCs	Variables		Calibration		Prediction		
				R_c_^2^	RMSECV (g kg^−1^)	RMSEC (g kg^−1^)	R_p_^2^	RMSEP (g kg^−1^)	RPD
Total saponins	Full-PLS	18	1577	0.9494	0.2340	0.1421	0.8506	0.1439	2.54
	CARS-PLS	16	66	0.9626	0.1691	0.1221	0.8949	0.1250	2.92
	UVE-PLS	17	872	0.9404	0.2622	0.1541	0.8210	0.1623	2.25
Mannitol	Full-PLS	18	1557	0.9716	0.3901	0.1914	0.9385	0.2876	4.01
	CARS-PLS	17	39	0.9868	0.1799	0.1307	0.9664	0.2192	5.26
	UVE-PLS	18	767	0.9722	0.3907	0.1894	0.9437	0.2795	4.13
Naringenin	Full-PLS	16	1557	0.8589	0.0041	0.002715	0.8432	0.003195	2.48
	CARS-PLS	17	28	0.8888	0.0031	0.002411	0.8570	0.003159	2.51
	UVE-PLS	18	256	0.8623	0.0040	0.002682	0.8326	0.003390	2.34

## Data Availability

The original contributions presented in the study are included in the article, further inquiries can be directed to the corresponding author.
